# Editorial: Technological innovations for improved prevention and diagnosis of oral disease

**DOI:** 10.3389/froh.2024.1481890

**Published:** 2024-09-16

**Authors:** Luis Felipe das Chagas e Silva de Carvalho, Rayssa Ferreira Zanatta

**Affiliations:** ^1^ Department of Dentistry, Universidade de Taubaté, Taubaté, Brazil; ^2^ Department of Dentistry, Universidade de Brasília, Brasilia, Brazil

**Keywords:** technology, dentistry, diagnosis, education, disease

**Editorial on the Research Topic**
Technological innovations for improved prevention and diagnosis of oral disease

Technological innovations are modulating and improving the field of dentistry, significantly enhancing the prevention, diagnosis, and treatment of oral diseases. From digital imaging tools to the integration of artificial intelligence, these advancements are enabling earlier and more accurate diagnoses, paving the way for less invasive and more effective treatments. Innovations in photonic applications, such as advanced light-based technologies, are driving the development of better diagnostic techniques and therapeutic appliances. The advent of scanning and 3D printing technologies is simplifying patient care, reducing costs, increasing precision, and enabling highly personalized treatment plans. Additionally, the rise of tele-dentistry, powered by internet and communication advancements, is extending dental care access to remote and underserved communities, democratizing access to oral healthcare. Smart devices, including apps and electronic toothbrushes, for example, are further enhancing daily dental care, empowering patients to maintain better oral health.

Building on the transformative impact of these technological advancements, the rapid and expansive growth of the global technology market is further accelerating progress in diagnostic and treatment methods across both medicine and dentistry. The importance of researchers being aware of new modalities for patient healthcare will facilitate the prospect for the development of new projects and studies that will directly benefit patients. A key factor in this ongoing evolution is the necessity of multidisciplinary collaboration. In today's research environment, the integration of diverse expertise—from clinical health professionals such as medical doctors, dentists, physiotherapists, and nurses, to specialists in biomedical engineering, systems and data analysis, and computer science—has become essential. This collaborative approach not only enriches the research process but also ensures that data analysis and technological integration are optimized, leading to more effective and comprehensive healthcare solutions.

The aforementioned point aligns with the studies developed in our research group, which is focused on integrating a cutting-edge real-time diagnostic technique utilizing body biofluids, such as saliva, urine or blood (1–3). This diagnostic technique is based on the use of infrared absorption spectroscopy with an attenuated total reflection accessory to detect possible biomarkers for several diseases, such as cancer, diabetes, among others. For that, we need to involve several professionals, and our multidisciplinary team exemplifies the power of collaboration, bringing together dentists with deep expertise in relevant pathologies, such as oral cancer, to guide the clinical application of this technology. Biomedical engineers and physicists contribute their specialized knowledge of the spectroscopic technique, ensuring its optimal function and continuous refinement. Meanwhile, professionals skilled in metadata analysis and programming are essential in developing and optimizing software that facilitates the real-time use of this diagnostic tool in clinical settings.

Form the dental perspective, studies involving salivary analysis have proven to be interesting from a clinical and scientific point of view, due to being easy to obtain and having a minimally (or not at all) invasive collection method. The evaluation of oral diseases such as cancer, caries, and periodontal disease can benefit from modern methods of early detection using saliva, as discussed in the edition. Further, assessment of the oral microbiome has allowed us to understand new nuances of these diseases and may, in the near future, guide more personalized treatment and control strategies.

Beyond enabling faster and more accurate diagnoses, as well as less invasive and longer-lasting treatments, technological advancements are also allowing the development of educational tools, greatly enhancing the accessibility and quality of information available, for students, clinicians and patients. Interactive platforms, virtual simulations, and online courses have made it possible for dental professionals to continuously update their skills and knowledge, while patients are empowered with reliable information to make informed decisions about their oral health. With the advancement of artificial intelligence, the challenge now will be to improve users’ critical skills.
